# Evaluation of the Relationship Between Aniseikonia and Stereopsis Using a New Method

**DOI:** 10.3389/fmed.2022.889398

**Published:** 2022-05-20

**Authors:** Lingxian Xu, Lu Liu, Huang Wu

**Affiliations:** Department of Optometry, The Second Hospital of Jilin University, Changchun, China

**Keywords:** stereopsis, induced aniseikonia, contour, random-dot, disparity, smartphone

## Abstract

**Purpose:**

To investigate the influence of induced aniseikonia on stereopsis measured by contour-based and random-dot-based stereograms using a new method.

**Methods:**

Unlike previous studies in which aniseikonia was induced using magnifiers, which potentially influenced the position of the test symbols in the half-view, here the image was magnified while maintaining each test symbol’s central position within the half-view. A phoropter and two 4K smartphones were used to measure stereopsis in seventeen young adults aged 20–28 years old. Stereopsis was tested using both contour-based and random-dot-based stereograms under overall or meridional aniseikonia with magnifications ranging from 2.5 to 30%. Repeated measures ANOVA was used to evaluate the effect of aniseikonia on stereopsis.

**Results:**

Stereopsis decreased with an increase in aniseikonia magnification in the overall, horizontal, and vertical directions. Stereopsis values (log arcsec) increased from 1.29 ± 0.14 at baseline to 2.38 ± 0.16 with 30% overall aniseikonia of contour-based stereograms. In random-dot based stereograms, stereopsis values increased from 1.29 ± 0.16 at baseline to 2.24 ± 0.23 with 22.5% overall aniseikonia. Overall aniseikonia caused a significantly greater impairment on stereopsis as compared with the changes in meridional directions. In contour-based stereograms, vertical aniseikonia had significantly less impact on stereopsis than horizontal aniseikonia of identical magnification. The opposite phenomenon was found in random-dot-based stereograms.

**Conclusion:**

Stereopsis decreased with an increase of magnification of induced aniseikonia. Magnifying patterns (overall, horizontal, or vertical) also significantly affected stereopsis. The conflicting impact of meridional aniseikonia on stereopsis measured by contour-based and random-dot-based stereograms may be associated with the uniqueness of the two test systems.

## Introduction

Aniseikonia is a condition where images seen with both eyes are perceived as being different in size and/or shape (1, 2). The possible causes of aniseikonia are optical, retinal, and cortical (3, 4). Optical aniseikonia is used to denote aniseikonia due to a physically measured difference in the sizes of the retinal images that typically arises in anisometropia, aphakia, and pseudophakia, among others (5, 6). Retinal aniseikonia may be due to the stretching or compression of the retina, leading to the alteration in spacing between the photoreceptors, which changes the perceived image size (1). Common causes include epiretinal membrane, macular edema, and central serous chorioretinopathy (7). Cortical aniseikonia may occur when apparent image sizes are perceived as differently due to abnormalities in the higher levels of visual processing system above beyond the retina (1, 3).

The apparent unequal size may be uniform (i.e., magnified or minified for all meridians). However, the size difference can also be meridional, wherein one image is larger or smaller in one specific meridian relative to the corresponding meridian in the other eye. This phenomenon can be observed when astigmatic anisometropia is present (1, 6). Discrepant image sizes may also be perceived by both eyes in retinal diseases such as epiretinal membrane (8) and macular edema (7).

Studies have suggested that the visual system can tolerate low amounts of aniseikonia without complete disruption of binocular fusion (9, 10). As the degree of aniseikonia increases, stereopsis becomes disrupted (11). Several studies discussed this issue by evaluating the effects of various degrees of induced aniseikonia on stereopsis, but the results varied widely, with stereopsis being reported to be perceived in aniseikonia of 4% or lower (4), 5% or lower (10), and 19% or lower (9). Most of these studies used size lenses to enlarge or reduce images in front of one eye. However, we observed that the enlargement of one eye’s image may introduce an additional disparity whether the size of the image is changed uniformly or solely in one meridian. This principle can be demonstrated with a four-circle test pattern like the Fly Stereo Acuity Test (Vision Assessment Corporation, Elk Grove Village, IL, United States).

If a size lens is placed in front of the left eye, then the left eye image is uniformly enlarged, whereas the right eye image is unchanged, resulting in aniseikonia. The locations of the centers of the circles viewed by the left eye are changed, whereas the locations of the centers of the circles viewed by the right eye are unchanged; this causes a shift in the position of the centers of the circles of the left eye. More specifically, in the left eye image, the center of the left circle would be shifted to the left for a certain distance, and the center of the right circle would be shifted to the right by the same distance. An uncrossed disparity would be created simply by moving the circle leftward in the left eye image, without considering the effect of magnification. Similarly, a crossed disparity would be created by moving the circle rightward in the left eye image, regardless of the effect of magnification. Consequently, four circles would appear at different depths; the circle on the left would seem farthest, the circle on the right side would seem nearest, and two circles located in the middle position would appear in the middle distance. In this situation, the participant’s ability to distinguish the stereo target is affected not only by the magnification effect over one eye, but also by the set disparities of the test material.

A similar situation exists in the random-dot test pattern such as “Pacman” symbol (TNO stereotest; Lameris Ootech BV, Ede, Netherlands). If a size lens is placed in front of the left eye, then the left eye image is uniformly enlarged, whereas the right eye image is unchanged. Consequently, uncrossed disparity would be created on the left side of the fused image, and crossed disparity would be created on the right side of the fused image. The additional induced disparities might rotate the random-dot pattern clockwise along the vertical axis. The “mouth” of the “Pacman” would be identified easily when facing left or right. However, the participant would experience difficulty in judging the orientation of the mouth when facing up or down because of the difficulty of dislocation fusion in the vertical direction.

In summary, the judgment of stereo symbols is complicated because of the newly introduced disparities by a lens that uniformly magnifies images. In order to minimize the introduction of additional disparities in the process of inducing aniseikonia, we adopted a new method to induce aniseikonia. The test image seen by the left eye was magnified. Four test symbols were utilized as conventional measurement methods (one out of four choose mode). For each test unit, the four symbols were arranged vertically in a line and each center of the four symbols was kept unchanged in the process of magnification. Meanwhile, the test image seen by the right eye was unchanged, so the location of each test symbol’s center viewed by the right eye was unchanged and the center of each test symbol’s pair still coincided in binocular view. Then we measured stereoacuity under induced aniseikonia. Since the interference of additional disparity was minimized as much as possible, the real effect of stereopsis by aniseikonia was evaluated.

Clinically, there are two commonly used stereopsis measurements: the contour-based stereograms (which are used to test local stereopsis) and random-dot-based stereograms (which are used to test global stereopsis). Some studies suggested that stereopsis values varied when measured by these two kinds of stereograms (12, 13). Lovasik et al. (14) reported a more rapid loss of stereoacuity with induced aniseikonia when measured by the contour stereogram relative to the loss measured by the random-dot stereogram. Therefore, in this study, the effect of aniseikonia on stereopsis was measured by both the contour-based and random-dot-based stereograms.

## Materials and Methods

### Participants

A total of 17 participants (5 men and 12 women) aged 20–28 years were recruited to this study. The best-corrected visual acuity of all participants was 0 logMAR or better. The stereothresholds of all participants were 40′′ or better, as measured using the Fly Stereo Acuity Test. All participants provided written informed consent before participating in the study. The research protocol observed the tenets of the Declaration of Helsinki and was approved by the ethics committee of the Second Hospital of Jilin University (No. 2020-110).

### Test System

#### Equipment

**FIGURE 1:**
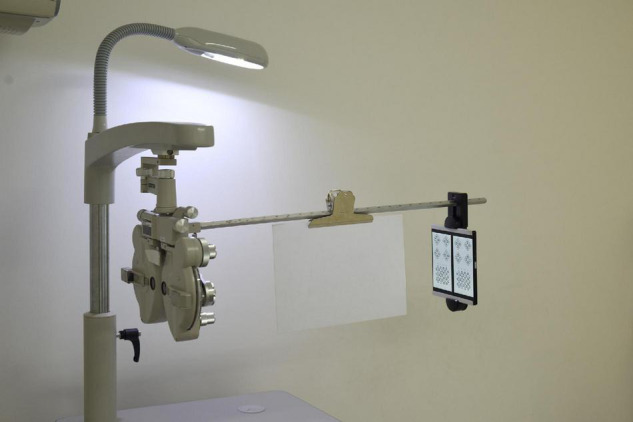
Photograph of the testing system.

A stereopsis measurement system was established with two 4K smartphones and a phoropter, as previously described (15–17). The resolution of the smartphone screen was 3840 × 2160 (Sony Xperia XZ Premium; Sony Mobile Communications Inc., Tokyo, Japan). With the aid of two approximately 5.5Δ based out Risley prisms, two smartphones are capable of creating a minimum 10′′ (1-pixel) disparity at a viewing distance of 0.65 m at the near-vision test rod of the phoropter (Topcon VT-10, Topcon Corp, Tokyo, Japan) (Figure 1).

#### Test Symbols

##### Contour-Based Symbols

The contour-based symbols were created to reproduce quantitative measurements using the Fly Stereo Acuity Test. However, the arrangement of the circles was modified in our testing regime. Specifically, four test circles were arranged vertically, and one of them was randomly chosen as the stereo target. The target circle appeared to stand out from the plane due to the crossed disparity. Three comparison circles were set along both sides of the test circles to maintain consistency with the Fly Stereo Acuity Test as much as possible. The participant identified the stereo target when the threshold of their stereopsis was lower than the setting disparity. In the newly designed test system, while magnifying the left eye image to induce aniseikonia, all test circles appeared to be rotated at a certain angle clockwise along the vertical axis of the screen. To minimize the influence of the rotation effect on the disparity evaluation, the test symbols in each test unit were arranged vertically.

##### Random-Dot-Based Symbols

The random-dot-based pattern comprised four squares composed of random dots arranged vertically. One circle was randomly hidden in one of the four squares. The participant was asked to determine the square comprising a circle protruding from the plane, which occurred when the participants’ stereopsis threshold was lower than the disparity of the depth-containing circle. The minimal size of the random dot was 6 × 6 pixels (equivalent to 0 logMAR resolution) to ensure that all of the dots could be distinguished by the participant (18).

#### Test Pages

##### Agreement Between Vertical and Conventional Arrangement Pattern

**FIGURE 2:**
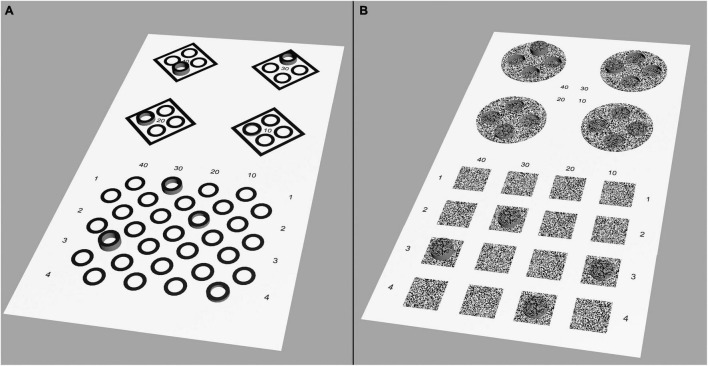
Simulation of the perceptions generated by the test images. If a patient’s stereothreshold is lower than the displayed disparity, the target appeared as protruding. **(A)** Contour-based pattern comparison test between four circles arranged in quadrilateral form and vertical line form. The disparity of the four circles arranged in both formations was 40′′, 30′′, 20′′, and 10′′, respectively. **(B)** Random-dot pattern comparison test between four circles arranged in quadrilateral and vertical line forms. The disparity of the four circles arranged in both forms was 40′′, 30′′, 20′′, and 10′′, respectively. In the quadrilateral arrangement, the other three circles had the same amount of disparity as the target but were designed to be uncrossed and appeared dented into the planes.

To test the agreement between the vertical arrangement pattern and the conventional arrangement of routine tests, we designed two tests including contour-based and random-dot-based patterns. In the contour-based test, the quantitative measurement section of the Fly Stereo Acuity Test was chosen for comparison (Figure 2A). In the random-dot-based test, the quantitative measurement section of the Random Dot 3 Stereo Acuity Test (Vision Assessment Corporation, Elk Grove Village, IL, United States) was chosen for comparison (Figure 2B).

##### Determining the Threshold of Stereopsis

Both the contour-based test and random-dot-based test used the same test pattern. There were two grade menus in the test system. The first grade menu comprised eight test units with a step range of 90′′, that is, 640′′, 550′′, 460′′, 370′′, 280′′, 190′′, 100′′, and 10′′. The second grade menu also comprised eight test units, but the step range was 10′′. There were seven test pages in the second grade menu (page 1, 20′′–90′′; page 2, 110′′–180′′; page 3, 200′′–270′′; page 4, 290′′–360′′; page 5, 380′′–450′′; page 6, 470′′–540′′; page 6, 560′′–630′′). A program written with C# was used to produce all stereograms with crossed disparity.

#### Aniseikonia Test Unit

**FIGURE 3:**
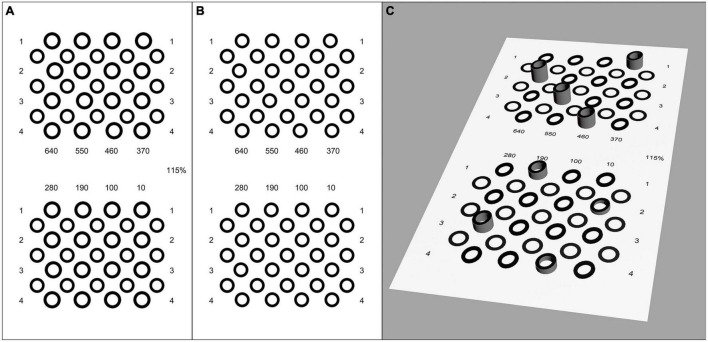
Legend of contour-based pattern of the first grade test page with 15% induced overall aniseikonia. **(A)** Left eye image. **(B)** Right eye image. **(C)** Simulation of the perception of the test images. The disparity of the target circle was 640′′, 550′′, 460′′, 370′′, 280′′, 190′′, 100′′, and 10′′. The center of the comparison circles was at the same point when fused correctly, but the size of test symbols in left eye image was enlarged by 15% because of the induced aniseikonia. All test circles appeared to be rotated at a certain angle clockwise along the vertical axis of the screen.

**FIGURE 4:**
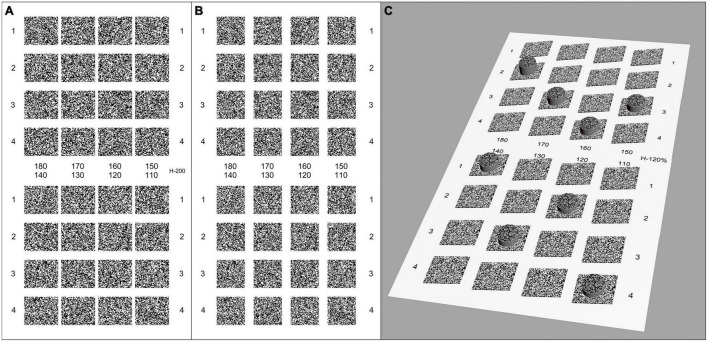
Legend of random-dot pattern of the second grade test page (110′′–180′′) with 20% induced horizontal aniseikonia. **(A)** Left eye image. **(B)** Right eye image. **(C)** Simulation of the perception of the test images. The disparity of the target circle was 180′′, 170′′, 160′′, 150′′, 140′′, 130′′, 120′′, and 110′′. The center of the comparison hidden circles was at the same point when fused correctly, but the size of test symbols in left eye image was enlarged by 20% in the horizontal direction to induce aniseikonia. All squares appeared to be rotated at a certain angle clockwise along the vertical axis of the screen.

To induce aniseikonia, the test image seen by the right eye was unchanged, and the test image seen by the left eye was enlarged to provide either overall, horizontal, or vertical magnification. Overall magnification enlarged the image consistently (Figure 3). Horizontal magnification only enlarged the image horizontally without enlarging the image vertically (Figure 4), while vertical magnification enlarged the image vertically without horizontal enlargement. The magnification rate ranged from 1.025 to 1.3, with a step range of 2.5%. The specific magnification rates were 1.025, 1.05, 1.075, 1.1, 1.125, 1.15, 1.175, 1.2, 1.225, 1.25, 1.275, and 1.3. While enlarging the test image, each test symbol was enlarged separately, with the location of its center unchanged in order to coincide the center of each test symbol’s pair on binocular view.

#### Test Procedure

##### Agreement Between Vertical and Conventional Arrangement Pattern

The participant was asked to distinguish the stereo circle from both the original pattern (40′′–10′′) and vertical arrangement pattern (40′′–10′′). The minimal stereo symbol that could be distinguished was recorded as the stereopsis threshold of the participant. The test sequence of contour-based and random-dot based stereograms was randomly determined.

##### Determining the Threshold of Stereopsis Under Induced Aniseikonia Conditions

**FIGURE 5:**
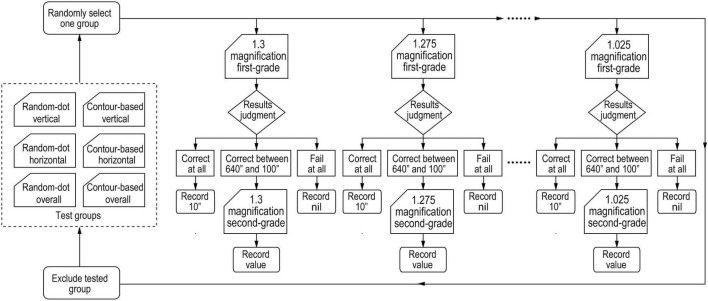
Flowchart of test procedure of determining the threshold for stereopsis under induced aniseikonia conditions. Test groups included three random-dot-based patterns (overall, horizontal, or vertical aniseikonia) and three contour-based patterns (overall, horizontal, or vertical aniseikonia). One group was selected randomly to test a participant. Two-step choices were conducted to measure the stereopsis threshold with a magnification of 1.3, and then 1.275, and so on, until it reached 1.025. Randomly, another group was chosen to do the examination again, and so on, until finishing all the six groups’ tests.

The test sequence of contour-based pattern or random-dot-based pattern was random. Random test sequences were also used for the overall, horizontal and vertical magnifications. A magnification of 1.3 was adopted at the beginning of the test, and then 1.275, 1.25, and so on, until it reached 1.025. At the beginning of the test, the first grade page was shown, and the participant was asked to find the stereo target from 640′′ to 10′′. If the participant pointed correctly at 10′′, then their stereopsis was recorded as 10′′. If the participant could point to the stereo target at 190′′ but failed to do so at 100′′, then the second page of the second grade menu (including 110′′–180′′) was chosen for the next examination. At this time, if the participant could ascertain the target circle at 160′′ but failed to do so at 150′′, then the stereopsis was recorded as 160′′. However, if they were unable to point to the stereo circle at 640′′, then the stereopsis was recorded as “nil.” Under this test procedure, the stereopsis of the participant could be determined from 10′′ to 640′′ with a measurement accuracy of 10′′ (Figure 5).

### Statistical Analysis

Statistical analysis was performed using SPSS (version 20.0; IBM Corp, Armonk, NY, United States) and GraphPad Prism (version 8.0.1; GraphPad Software Inc., San Diego, CA, United States). The Wilcoxon signed-rank test and Kendall’s coefficient of concordance (Kendall’s W) were conducted to examine the agreement between vertical and conventional arrangement patterns. Stereopsis values under aniseikonia were transformed to log arcsec values for analysis. The normality of the distribution of the stereoacuity (log arcsec) was checked by the D’Agostino and Pearson test, and 64 of 69 sets of data were normally distributed. On this basis, repeated measures ANOVA was applied to determine significant main effects and interactions at *p* = 0.05. Partial eta-squared (η_*p*_^2^) was calculated as an effect size measure. Greenhouse-Geisser correction was used if the assumption of sphericity was not met, and the Bonferroni test was used in *post-hoc* analysis. Minitab Statistical Software (version 19; Minitab Inc., State College, PA, United States) was used to compare the slopes of the change in stereopsis with induced aniseikonia for the contour-based and random-dot based stereograms.

## Results

### Agreement Between Vertical and Conventional Arrangement Patterns

The Wilcoxon signed-rank test showed that there was no significant difference between the stereopsis threshold measured by vertical and conventional arrangement patterns (*Z* = −0.577, *P* = 0.564 in both contour-based and random-dot-based stereograms). Kendall W tests showed a significant agreement between stereopsis threshold measured by vertical and conventional arrangement patterns (Kendall *W* = 0.853, *P* = 0.038 in contour-based stereograms; Kendall *W* = 0.927, *P* = 0.020 in random-dot-based stereograms).

### Stereopsis Changes With Different Aniseikonia

**FIGURE 6:**
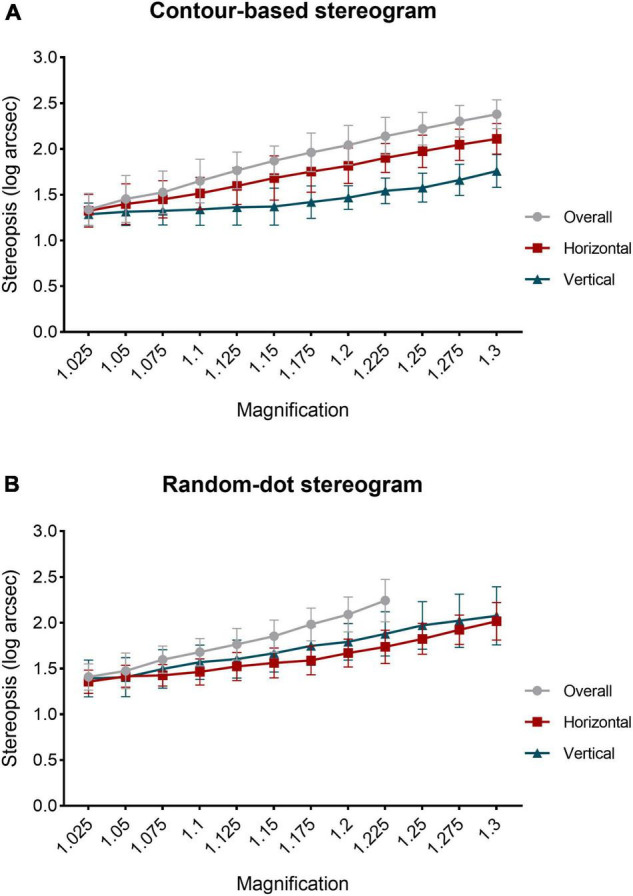
Relationship between induced aniseikonia and stereopsis values (log arcsec). **(A)** Contour-based stereograms. **(B)** Random-dot-based stereograms. Datapoints and error bars represent mean with standard deviation (SD). The final three data points of overall magnification curve in the random-dot stereogram image were not plotted because some participants failed to pass the test when magnification increased to 25, 27.5, and 30%.

Raw stereopsis values under different conditions of aniseikonia are provided in Supplementary Tables 1–7. Stereopsis values were transformed to log arcsec for analysis. The mean ± standard deviation (SD) of stereopsis values (log arcsec) for baseline and each aniseikonia condition for contour-based and random-dot-based stereograms are shown in Table 1. The stereopsis values increased with the increase in aniseikonia magnification; this was observed in the overall, horizontal, and vertical directions, in both stereogram types (Figure 6). In contour-based stereograms, stereopsis was present until the magnification increased to 30% for overall, horizontal, and vertical patterns. The stereopsis values (log arcsec) at baseline averaged 1.29 and increased to 2.38 with 30% overall aniseikonia. In random-dot-based stereograms, for overall aniseikonia, stereoacuity was measured for magnifications ranging from 2.5 to 22.5%; several of the participants (7/17) failed the stereopsis test under 25%; most participants (12/17) failed the test under 27.5%, and all participants failed the test under 30%. In horizontal and vertical aniseikonia, stereoacuity could be measured under all magnifications ranging from 2.5 to 30%. The stereopsis values (log arcsec) at baseline averaged 1.29 and increased to 2.24 with 22.5% overall aniseikonia.

**TABLE 1:** Mean ± SD of stereopsis values (log arcsec) tested under different conditions of aniseikonia.

Magnification	Contour-based stereograms			Random-dot-based stereograms		
		
	Overall	Horizontal	Vertical	Overall	Horizontal	Vertical
1	1.29 ± 0.14			1.29 ± 0.16		
1.025	1.34 ± 0.18	1.32 ± 0.18	1.29 ± 0.12	1.41 ± 0.14	1.36 ± 0.13	1.39 ± 0.20
1.05	1.45 ± 0.26	1.40 ± 0.22	1.31 ± 0.15	1.48 ± 0.19	1.42 ± 0.12	1.41 ± 0.21
1.075	1.53 ± 0.23	1.45 ± 0.20	1.32 ± 0.16	1.60 ± 0.15	1.43 ± 0.12	1.50 ± 0.21
1.1	1.65 ± 0.24	1.51 ± 0.17	1.34 ± 0.18	1.68 ± 0.15	1.46 ± 0.14	1.57 ± 0.19
1.125	1.77 ± 0.20	1.59 ± 0.20	1.36 ± 0.20	1.77 ± 0.17	1.52 ± 0.15	1.60 ± 0.21
1.15	1.87 ± 0.16	1.68 ± 0.24	1.37 ± 0.20	1.85 ± 0.18	1.56 ± 0.16	1.67 ± 0.21
1.175	1.96 ± 0.21	1.75 ± 0.22	1.42 ± 0.18	1.98 ± 0.18	1.59 ± 0.16	1.75 ± 0.21
1.2	2.04 ± 0.21	1.82 ± 0.19	1.47 ± 0.13	2.09 ± 0.19	1.67 ± 0.15	1.79 ± 0.20
1.225	2.14 ± 0.20	1.90 ± 0.16	1.54 ± 0.14	2.24 ± 0.23	1.74 ± 0.18	1.88 ± 0.24
1.25	2.22 ± 0.18	1.97 ± 0.18	1.58 ± 0.16	–	1.82 ± 0.17	1.97 ± 0.26
1.275	2.30 ± 0.17	2.05 ± 0.17	1.66 ± 0.17	–	1.92 ± 0.16	2.02 ± 0.29
1.3	2.38 ± 0.16	2.11 ± 0.17	1.76 ± 0.18	–	2.02 ± 0.20	2.08 ± 0.32

### Differences Between Overall, Horizontal, and Vertical Aniseikonia

Repeated measures ANOVA was conducted with magnification and magnifying pattern (overall, horizontal, or vertical) as within-subject variables. In contour-based stereograms with aniseikonia (2.5–30%), a significant main effect of magnification (*F*_(3,48)_ = 190.91, *P* < 0.001, η_*p*_^2^ = 0.923), of magnifying pattern (*F*_(1,23)_ = 163.31, *P* < 0.001, η_*p*_^2^ = 0.911) and a significant interaction between magnification and magnifying pattern (*F*_(6,96)_ = 22.42, *P* < 0.001, η_*p*_^2^ = 0.584) was found. Bonferroni’s *post-hoc* test showed that stereoacuity under each magnifying pattern was significantly different from the other two patterns in identical aniseikonia from 10 to 30% (*P* < 0.001 to *P* = 0.015).

In random-dot-based stereograms with aniseikonia (2.5–22.5%), a significant main effect of magnification (*F*_(2,40)_ = 162.30, *P* < 0.001, η_*p*_^2^ = 0.910), of magnifying pattern (*F*_(2,31)_ = 34.38, *P* < 0.001, η_*p*_^2^ = 0.682) and a significant interaction between magnification and magnifying pattern (*F*_(6,102)_ = 17.18, *P* < 0.001, η_*p*_^2^ = 0.518) was observed. Bonferroni’s *post-hoc* test showed that stereopsis values (log arcsec) were significantly higher for overall aniseikonia than for meridional aniseikonia, ranging between 7.5 and 22.5% (*P* < 0.001 to *P* = 0.026). Vertical aniseikonia values of 15, 17.5, and 22.5% yielded significantly (*P* = 0.003 to *P* = 0.047) higher stereothresholds compared with the same values of horizontal aniseikonia.

### Differences Between Contour-Based and Random-Dot-Based Stereograms

Repeated measures ANOVA was conducted with magnification and test method (contour-based stereogram, random-dot-based stereogram) as within-subject variables. For overall aniseikonia (2.5–22.5%), there was a significant main effect of magnification (*F*_(4,58)_ = 201.92, *P* < 0.001, η_*p*_^2^ = 0.927). However, there was no significant main effect of test method and no significant interaction between magnification and test method.

For horizontal aniseikonia, there was a significant main effect of magnification (*F*_(4,60)_ = 190.31, *P* < 0.001, η_*p*_^2^ = 0.922), of test method (*F*_(1,16)_ = 6.19, *P* = 0.024, η_*p*_^2^ = 0.279), and a significant interaction between magnification and test method (*F*_(5,76)_ = 4.96, *P* = 0.001, η_*p*_^2^ = 0.237). Bonferroni’s *post-hoc* test showed that stereopsis values (log arcsec) under horizontal aniseikonia of 17.5, 20, and 22.5% measured by contour-based stereograms was significantly higher than that of random-dot-based stereograms (*P* = 0.016 to *P* = 0.045).

For vertical aniseikonia, a significant main effect of magnification (*F*_(3,42)_ = 95.79, *P* < 0.001, η_*p*_^2^ = 0.857), of test method (*F*_(1,16)_ = 64.07, *P* < 0.001, η_*p*_^2^ = 0.800), and a significant interaction between magnification and test method (*F*_(3,41)_ = 8.65, *P* < 0.001, η_*p*_^2^ = 0.351) was found. Bonferroni’s *post-hoc* test showed that stereopsis values (log arcsec) measured under conditions of induced vertical aniseikonia ranging from 7.5 to 30% with contour-based stereograms were lower than those measured using random-dot-based stereograms (*P* < 0.001 to *P* = 0.008).

For overall aniseikonia, there was no significant difference of the linear regression slopes for stereoacuity over magnification between two test methods (contour-based stereograms slope 3.82; random-dot-based stereograms slope 4.09; *P* = 0.350). For horizontal aniseikonia, the slope of the regression line of the contour-based stereograms was significantly higher than that of random-dot-based stereograms (2.91 for the contour-based stereograms; 2.30 for the random-dot-based stereograms; *P* = 0.003). For vertical aniseikonia, the slope of the regression line of the contour-based stereograms was significantly lower than that of the random-dot-based stereograms (1.60 for the contour-based stereograms; 2.60 for the random-dot-based stereograms; *P* < 0.001).

## Discussion

The relationship between induced aniseikonia and stereopsis has been investigated for decades (9, 10, 19–21). Highman (9) examined 30 participants with different degrees of myopia. Each participant wore a contact lens in the eye with a smaller diopter and wore the appropriate correcting lens in a trial frame over the more myopic eye to induce aniseikonia of various magnitudes. The Titmus Stereo-circles Chart was used to test stereopsis under different magnifications of aniseikonia. Findings indicated that stereopsis can be measured even with 19% of induced aniseikonia. Oguchi and Mashima (10) investigated the impact of artificial aniseikonia on binocular vision both psychophysically and objectively. They used a random-dot stereogram to measure stereoacuity and visual evoked potential under conditions of aniseikonia induced by size lenses ranging from 2 to 15% in six participants with normal stereoacuity. Their findings showed that stereopsis could be perceived with aniseikonia of 5% or lower. Moreover, they found a declining trend in the amplitude of visual evoked potential as the aniseikonia increased.

The measurement of stereoacuity might be interfered by the additional disparities introduced by size lenses that uniformly magnifies images. To minimize the interference of additional disparities in the process of inducing aniseikonia, the images used in our study were magnified while maintaining the position of each symbol’s center. Then the stereoacuity was measured under induced aniseikonia so that the real effect of aniseikonia on stereopsis could be accessed with the minimal interference of additional disparities. The consistency between the vertical and conventional arrangement patterns was first evaluated to ensure the possibility of substitution. There was a high degree of agreement between the vertical and conventional arrangement stereograms in both contour-based and random-dot-based stereograms.

In our study, stereopsis declined with an increase in aniseikonia magnification measured by both contour-based and random-dot-based stereograms. This is consistent with previous studies (3, 14, 22). Atchison et al. (3) used afocal magnification lenses of 3, 6, 9, and 12% to induce aniseikonia. Stereoacuity was tested using random-dot stereograms with a “Pacman” shape. They transformed the stereoacuity to log values and found that the threshold of stereopsis increased with an increase in aniseikonia, and the loss was approximately proportional to the square of aniseikonia. Lovasik and Szymkiw (14) induced aniseikonia using magnifiers of 26 magnifications ranging from 1.2 to 32.3% and measured stereoacuity using both the Titmus stereo test and the Randot test. Their results indicated that the value of stereopsis (arcsec) increased with increasing aniseikonia in a curvilinear manner.

In our study, the effect of overall aniseikonia was always larger than that of meridional aniseikonia of identical magnification. This was similar to the results of Atchison et al. (3), who found that the mean loss of stereopsis with meridional aniseikonia was approximately 64% of that for overall aniseikonia with identical magnification.

In this study, participants’ response to contour-based and random-dot-based stereograms was different in relatively high aniseikonia. For overall aniseikonia, the stereopsis still presented up to 30% when testing with contour-based stereograms, whereas, some participants failed at magnifications of 25, 27.5, and 30% when tested with random-dot-based stereograms.

In the contour-based stereogram, the effect of vertical aniseikonia was significantly smaller than that of horizontal aniseikonia of identical magnification. In random-dot-based stereograms, the opposite trend was found in three out of nine magnifications (15, 17.5, and 22.5%). This difference may be related to variations in the characteristics of the two test targets. Stereopsis is affected primarily in the horizontal meridian. In contour-based stereograms, image matching between the two eyes is determined by the contour. When the vertical direction of the half-view of one eye is enlarged with respect to the fellow half-view, the horizontal direction remains unchanged, which could help participants to distinguish the target symbols more easily. Conversely, when the horizontal direction of the half-view of one eye is enlarged with respect to the fellow half-view, the position of the lines in the horizontal direction changed simultaneously; this is likely to have a greater effect on depth perception and may explain why stereopsis was better under vertical, as opposed to horizontal aniseikonia. Whereas in random-dot-based stereograms, the participant is required to match dense random dots between the left and right eyes and fuse them into a single image. Since vertical disparities are less tolerated by the visual system, the processing capacity of fusing is more powerful in the horizontal than in the vertical direction (23, 24). Thus, it was easier for our participants to fuse images when enlarging one image in the horizontal direction than when enlarging one image in the vertical direction.

This study had some limitations. The participants that were recruited were young and had good stereoacuity; they therefore cannot accurately represent the general population. Induced aniseikonia may differ from that experienced by individuals with actual aniseikonia, and the adaptation of aniseikonia was not considered in this study. Besides, this test could not be achieved in a real circumstance to induce aniseikonia like size lens. It was carried out under an artificial test condition which is partly different from the actual situation of aniseikonia that occurred in the clinic. Moreover, the interference of additional disparities was minimized but was not eliminated completely.

## Conclusion

In this study, stereopsis decreased with increasing aniseikonia induced by both overall and meridional magnification. Overall aniseikonia decreased stereopsis more than meridional aniseikonia of similar magnitudes. Horizontal aniseikonia impaired the stereopsis of the contour-based stereograms significantly more than random-dot-based stereograms, whereas vertical aniseikonia impaired the stereopsis of random-dot-based stereograms significantly more than the contour-based stereograms.

## Data Availability Statement

The original contributions presented in the study are included in the article/Supplementary Material, further inquiries can be directed to the corresponding author.

## Ethics Statement

The studies involving human participants were reviewed and approved by the Ethics Committee of The Second Hospital of Jilin University (No. 2020-110). The patients/participants provided their written informed consent to participate in this study.

## Author Contributions

LX: writing the manuscript, conducting the experiment, and data collection. LL: material preparation and data analysis. HW: design the experiment, material preparation, and revision of the manuscript. All authors contributed to the article and approved the submitted version.

## Conflict of Interest

The authors declare that the research was conducted in the absence of any commercial or financial relationships that could be construed as a potential conflict of interest.

## Publisher’s Note

All claims expressed in this article are solely those of the authors and do not necessarily represent those of their affiliated organizations, or those of the publisher, the editors and the reviewers. Any product that may be evaluated in this article, or claim that may be made by its manufacturer, is not guaranteed or endorsed by the publisher.
