# Supplementary material for: Evaluation of the Relationship Between Aniseikonia and Stereopsis Using a New Method
Source: Front Med (Lausanne). 2022 May 20;9:889398. doi: 10.3389/fmed.2022.889398 (PMC9163365; doi:10.3389/fmed.2022.889398)
Supplement: Supplementary file 1 [file Data_Sheet_1.docx]

***Supplementary Material***

**Supplementary Table 1.**

**Test results of contour-based stereopsis values (arcsec) under overall aniseikonia**

| ID | Magnification | | | | | | | | | | | |
| --- | --- | --- | --- | --- | --- | --- | --- | --- | --- | --- | --- | --- |
|  | 1.025 | 1.05 | 1.075 | 1.1 | 1.125 | 1.15 | 1.175 | 1.2 | 1.225 | 1.25 | 1.275 | 1.3 |
| 1 | 10 | 10 | 10 | 10 | 20 | 40 | 40 | 50 | 60 | 80 | 130 | 140 |
| 2 | 30 | 30 | 30 | 40 | 70 | 70 | 70 | 80 | 90 | 90 | 160 | 170 |
| 3 | 20 | 50 | 60 | 60 | 60 | 60 | 60 | 60 | 70 | 80 | 80 | 170 |
| 4 | 50 | 50 | 50 | 50 | 50 | 70 | 70 | 110 | 130 | 140 | 180 | 180 |
| 5 | 10 | 10 | 20 | 40 | 50 | 60 | 70 | 90 | 140 | 140 | 170 | 240 |
| 6 | 20 | 20 | 40 | 60 | 80 | 80 | 150 | 160 | 200 | 200 | 320 | 320 |
| 7 | 20 | 30 | 40 | 40 | 60 | 60 | 60 | 70 | 140 | 170 | 170 | 190 |
| 8 | 20 | 50 | 50 | 50 | 60 | 80 | 120 | 140 | 150 | 160 | 170 | 170 |
| 9 | 20 | 40 | 40 | 50 | 60 | 80 | 170 | 170 | 230 | 240 | 250 | 420 |
| 10 | 20 | 20 | 20 | 20 | 30 | 40 | 50 | 60 | 70 | 170 | 230 | 260 |
| 11 | 20 | 20 | 20 | 40 | 40 | 60 | 70 | 80 | 80 | 140 | 140 | 160 |
| 12 | 20 | 40 | 40 | 50 | 80 | 130 | 130 | 170 | 210 | 230 | 280 | 380 |
| 13 | 30 | 30 | 40 | 50 | 50 | 70 | 70 | 70 | 140 | 160 | 170 | 190 |
| 14 | 30 | 30 | 40 | 110 | 140 | 150 | 170 | 200 | 230 | 290 | 320 | 350 |
| 15 | 40 | 100 | 100 | 110 | 120 | 130 | 200 | 220 | 220 | 230 | 320 | 350 |
| 16 | 20 | 20 | 30 | 40 | 70 | 100 | 140 | 190 | 180 | 200 | 220 | 280 |
| 17 | 20 | 20 | 20 | 40 | 50 | 70 | 100 | 170 | 240 | 330 | 350 | 350 |

**Supplementary Table 2.**

**Test results of contour-based stereopsis values (arcsec) under horizontal aniseikonia**

| ID | Magnification | | | | | | | | | | | |
| --- | --- | --- | --- | --- | --- | --- | --- | --- | --- | --- | --- | --- |
|  | 1.025 | 1.05 | 1.075 | 1.1 | 1.125 | 1.15 | 1.175 | 1.2 | 1.225 | 1.25 | 1.275 | 1.3 |
| 1 | 10 | 10 | 10 | 20 | 20 | 20 | 30 | 40 | 50 | 50 | 60 | 60 |
| 2 | 20 | 20 | 30 | 30 | 30 | 60 | 70 | 90 | 90 | 120 | 120 | 140 |
| 3 | 20 | 20 | 20 | 20 | 20 | 20 | 30 | 40 | 60 | 70 | 70 | 80 |
| 4 | 40 | 40 | 40 | 40 | 40 | 40 | 60 | 70 | 80 | 100 | 100 | 180 |
| 5 | 10 | 10 | 20 | 30 | 40 | 50 | 60 | 80 | 100 | 130 | 130 | 150 |
| 6 | 20 | 30 | 50 | 50 | 60 | 60 | 60 | 60 | 70 | 140 | 150 | 150 |
| 7 | 20 | 20 | 30 | 30 | 40 | 60 | 60 | 70 | 80 | 80 | 80 | 80 |
| 8 | 20 | 40 | 40 | 40 | 50 | 50 | 60 | 70 | 110 | 130 | 140 | 140 |
| 9 | 20 | 30 | 30 | 40 | 80 | 80 | 80 | 80 | 80 | 80 | 140 | 230 |
| 10 | 20 | 20 | 20 | 20 | 20 | 20 | 20 | 30 | 50 | 50 | 50 | 80 |
| 11 | 20 | 20 | 20 | 20 | 30 | 40 | 40 | 40 | 40 | 50 | 80 | 100 |
| 12 | 20 | 30 | 30 | 40 | 40 | 40 | 90 | 90 | 110 | 140 | 140 | 140 |
| 13 | 20 | 20 | 20 | 20 | 30 | 30 | 30 | 40 | 70 | 100 | 100 | 110 |
| 14 | 50 | 50 | 50 | 60 | 60 | 110 | 110 | 140 | 150 | 170 | 170 | 180 |
| 15 | 40 | 70 | 70 | 70 | 100 | 110 | 130 | 130 | 130 | 130 | 220 | 230 |
| 16 | 20 | 30 | 30 | 40 | 40 | 100 | 100 | 100 | 110 | 120 | 130 | 140 |
| 17 | 20 | 20 | 20 | 30 | 40 | 50 | 50 | 50 | 60 | 60 | 140 | 150 |

**Supplementary Table 3.**

**Test results of contour-based stereopsis values (arcsec) under vertical aniseikonia**

| ID | Magnification | | | | | | | | | | | |
| --- | --- | --- | --- | --- | --- | --- | --- | --- | --- | --- | --- | --- |
|  | 1.025 | 1.05 | 1.075 | 1.1 | 1.125 | 1.15 | 1.175 | 1.2 | 1.225 | 1.25 | 1.275 | 1.3 |
| 1 | 10 | 10 | 10 | 10 | 10 | 10 | 10 | 20 | 20 | 20 | 20 | 30 |
| 2 | 20 | 20 | 20 | 20 | 20 | 20 | 30 | 30 | 30 | 30 | 30 | 30 |
| 3 | 20 | 20 | 20 | 20 | 20 | 20 | 20 | 20 | 20 | 20 | 30 | 60 |
| 4 | 20 | 20 | 20 | 20 | 20 | 20 | 20 | 20 | 30 | 40 | 40 | 40 |
| 5 | 10 | 10 | 10 | 10 | 10 | 10 | 20 | 20 | 20 | 20 | 30 | 40 |
| 6 | 20 | 20 | 20 | 20 | 20 | 20 | 30 | 30 | 30 | 30 | 50 | 50 |
| 7 | 20 | 20 | 20 | 20 | 20 | 20 | 20 | 20 | 30 | 40 | 50 | 70 |
| 8 | 20 | 30 | 30 | 30 | 30 | 30 | 30 | 30 | 40 | 40 | 50 | 50 |
| 9 | 20 | 20 | 40 | 40 | 40 | 40 | 40 | 40 | 50 | 70 | 80 | 100 |
| 10 | 20 | 20 | 20 | 20 | 20 | 20 | 20 | 30 | 40 | 40 | 40 | 50 |
| 11 | 20 | 20 | 20 | 20 | 30 | 40 | 40 | 40 | 50 | 50 | 60 | 60 |
| 12 | 20 | 20 | 20 | 20 | 20 | 20 | 30 | 30 | 40 | 50 | 50 | 90 |
| 13 | 20 | 20 | 20 | 20 | 20 | 20 | 20 | 30 | 40 | 50 | 50 | 50 |
| 14 | 20 | 30 | 30 | 40 | 50 | 50 | 50 | 50 | 50 | 50 | 50 | 50 |
| 15 | 30 | 40 | 30 | 30 | 40 | 40 | 40 | 40 | 50 | 50 | 110 | 130 |
| 16 | 30 | 30 | 30 | 40 | 40 | 40 | 40 | 40 | 40 | 40 | 50 | 100 |
| 17 | 20 | 20 | 20 | 20 | 20 | 20 | 20 | 30 | 40 | 40 | 50 | 60 |

**Supplementary Table 4.**

**Test results of random-dot-based stereopsis values (arcsec) under overall aniseikonia**

| ID | Magnification | | | | | | | | | | | |
| --- | --- | --- | --- | --- | --- | --- | --- | --- | --- | --- | --- | --- |
|  | 1.025 | 1.05 | 1.075 | 1.1 | 1.125 | 1.15 | 1.175 | 1.2 | 1.225 | 1.25 | 1.275 | 1.3 |
| 1 | 20 | 30 | 30 | 30 | 30 | 40 | 50 | 80 | 80 | 90 | 110 | - |
| 2 | 20 | 20 | 30 | 40 | 50 | 50 | 60 | 60 | 110 | 130 | 170 | - |
| 3 | 20 | 20 | 40 | 50 | 60 | 60 | 70 | 80 | 150 | 160 | - | - |
| 4 | 20 | 20 | 30 | 30 | 40 | 40 | 100 | 280 | 280 | 280 | 280 | - |
| 5 | 20 | 20 | 20 | 30 | 40 | 50 | 60 | 70 | 80 | 90 | 90 | - |
| 6 | 20 | 30 | 50 | 50 | 110 | 110 | 130 | 140 | 150 | 260 | - | - |
| 7 | 40 | 60 | 60 | 60 | 70 | 80 | 90 | 130 | 150 | 170 | 300 | - |
| 8 | 40 | 40 | 40 | 60 | 80 | 90 | 90 | 150 | 150 | 340 | - | - |
| 9 | 30 | 30 | 40 | 40 | 50 | 60 | 110 | 110 | 320 | 520 | - | - |
| 10 | 30 | 60 | 60 | 80 | 80 | 120 | 130 | 150 | 340 | - | - | - |
| 11 | 30 | 40 | 50 | 80 | 130 | 150 | 150 | 200 | 420 | - | - | - |
| 12 | 20 | 20 | 40 | 40 | 40 | 50 | 110 | 110 | 160 | - | - | - |
| 13 | 20 | 20 | 30 | 40 | 50 | 70 | 80 | 90 | 160 | - | - | - |
| 14 | 40 | 60 | 70 | 80 | 80 | 110 | 200 | 200 | 400 | - | - | - |
| 15 | 50 | 50 | 60 | 70 | 70 | 110 | 200 | 240 | 240 | - | - | - |
| 16 | 20 | 20 | 30 | 40 | 50 | 60 | 70 | 110 | 110 | - | - | - |
| 17 | 20 | 20 | 30 | 40 | 40 | 60 | 70 | 100 | 110 | 160 | - | - |

**Supplementary Table 5.**

**Test results of random-dot-based stereopsis values (arcsec) under horizontal aniseikonia**

| ID | Magnification | | | | | | | | | | | |
| --- | --- | --- | --- | --- | --- | --- | --- | --- | --- | --- | --- | --- |
|  | 1.025 | 1.05 | 1.075 | 1.1 | 1.125 | 1.15 | 1.175 | 1.2 | 1.225 | 1.25 | 1.275 | 1.3 |
| 1 | 20 | 20 | 20 | 20 | 20 | 20 | 20 | 30 | 30 | 30 | 40 | 40 |
| 2 | 20 | 30 | 30 | 40 | 40 | 50 | 50 | 60 | 70 | 80 | 90 | 130 |
| 3 | 20 | 20 | 20 | 20 | 20 | 30 | 30 | 40 | 40 | 50 | 60 | 70 |
| 4 | 20 | 30 | 30 | 50 | 60 | 60 | 60 | 60 | 70 | 70 | 80 | 80 |
| 5 | 10 | 20 | 20 | 20 | 20 | 20 | 20 | 30 | 40 | 50 | 70 | 80 |
| 6 | 20 | 20 | 20 | 20 | 30 | 30 | 40 | 50 | 50 | 50 | 60 | 110 |
| 7 | 30 | 40 | 40 | 50 | 60 | 70 | 70 | 80 | 110 | 120 | 130 | 170 |
| 8 | 30 | 40 | 40 | 40 | 40 | 40 | 40 | 50 | 60 | 60 | 60 | 60 |
| 9 | 30 | 30 | 30 | 30 | 40 | 50 | 60 | 70 | 130 | 140 | 160 | 210 |
| 10 | 30 | 30 | 30 | 30 | 30 | 30 | 30 | 30 | 50 | 60 | 60 | 80 |
| 11 | 30 | 30 | 30 | 30 | 30 | 40 | 40 | 50 | 60 | 80 | 110 | 240 |
| 12 | 20 | 20 | 20 | 30 | 40 | 40 | 40 | 40 | 50 | 70 | 110 | 110 |
| 13 | 20 | 20 | 20 | 20 | 30 | 30 | 30 | 30 | 30 | 60 | 80 | 110 |
| 14 | 30 | 40 | 40 | 40 | 40 | 50 | 50 | 70 | 70 | 110 | 130 | 180 |
| 15 | 30 | 30 | 30 | 30 | 40 | 40 | 40 | 50 | 50 | 50 | 120 | 120 |
| 16 | 20 | 20 | 30 | 30 | 40 | 40 | 50 | 70 | 70 | 90 | 90 | 100 |
| 17 | 20 | 20 | 20 | 20 | 20 | 20 | 30 | 30 | 30 | 50 | 70 | 70 |

**Supplementary Table 6.**

**Test results of random-dot-based stereopsis values (arcsec) under vertical aniseikonia**

| ID | Magnification | | | | | | | | | | | |
| --- | --- | --- | --- | --- | --- | --- | --- | --- | --- | --- | --- | --- |
|  | 1.025 | 1.05 | 1.075 | 1.1 | 1.125 | 1.15 | 1.175 | 1.2 | 1.225 | 1.25 | 1.275 | 1.3 |
| 1 | 20 | 20 | 20 | 20 | 20 | 20 | 20 | 20 | 20 | 20 | 20 | 20 |
| 2 | 20 | 20 | 30 | 30 | 30 | 40 | 40 | 50 | 50 | 50 | 50 | 50 |
| 3 | 20 | 20 | 30 | 30 | 30 | 40 | 40 | 40 | 40 | 50 | 50 | 50 |
| 4 | 20 | 20 | 20 | 30 | 50 | 60 | 70 | 70 | 80 | 190 | 280 | 280 |
| 5 | 10 | 10 | 10 | 20 | 20 | 20 | 20 | 30 | 40 | 60 | 60 | 70 |
| 6 | 20 | 20 | 20 | 30 | 30 | 50 | 50 | 50 | 50 | 50 | 50 | 50 |
| 7 | 40 | 40 | 50 | 60 | 70 | 70 | 80 | 80 | 120 | 130 | 140 | 170 |
| 8 | 30 | 40 | 40 | 50 | 50 | 60 | 70 | 70 | 70 | 90 | 140 | 150 |
| 9 | 40 | 50 | 50 | 50 | 50 | 70 | 80 | 80 | 160 | 160 | 160 | 270 |
| 10 | 80 | 80 | 80 | 80 | 80 | 80 | 110 | 140 | 140 | 140 | 150 | 150 |
| 11 | 30 | 30 | 40 | 80 | 100 | 100 | 110 | 110 | 150 | 200 | 230 | 230 |
| 12 | 20 | 20 | 40 | 40 | 40 | 50 | 60 | 60 | 90 | 110 | 110 | 110 |
| 13 | 20 | 20 | 20 | 20 | 20 | 20 | 50 | 60 | 60 | 150 | 150 | 200 |
| 14 | 20 | 20 | 40 | 40 | 50 | 50 | 80 | 80 | 100 | 110 | 150 | 200 |
| 15 | 40 | 40 | 40 | 40 | 40 | 50 | 50 | 70 | 110 | 110 | 140 | 150 |
| 16 | 20 | 20 | 30 | 40 | 50 | 50 | 60 | 70 | 80 | 100 | 110 | 170 |
| 17 | 20 | 20 | 30 | 30 | 30 | 40 | 60 | 70 | 100 | 110 | 140 | 150 |

**Supplementary Table 7.**

**Median (interquartile range) of stereopsis values tested under different conditions of aniseikonia**

|  | Contour-based stereograms | | | Random-dot-based stereograms | | |
| --- | --- | --- | --- | --- | --- | --- |
| Magnification | Overall | Horizontal | Vertical | Overall | Horizontal | Vertical |
| 1 | 20(20,20) | | | 20(20,25) | | |
| 1.025 | 20(20,30) | 20(20,20) | 20(20,20) | 20(20,35) | 20(20,30) | 20(20,35) |
| 1.05 | 30(20,45) | 20(20,35) | 20(20,25) | 30(20,45) | 30(20,30) | 20(20,40) |
| 1.075 | 40(20,45) | 30(20,40) | 20(20,30) | 40(30,55) | 30(20,30) | 30(20,40) |
| 1.1 | 50(40,55) | 30(20,40) | 20(20,30) | 40(40,65) | 30(20,40) | 40(30,50) |
| 1.125 | 60(50,75) | 40(30,55) | 20(20,35) | 50(40,80) | 40(25,40) | 40(30,50) |
| 1.15 | 70(60,90) | 50(35,70) | 20(20,40) | 60(50,110) | 40(30,50) | 50(40,65) |
| 1.175 | 70(65,145) | 60(35,85) | 30(20,40) | 90(70,130) | 40(30,50) | 60(45,80) |
| 1.2 | 110(70,170) | 70(40,90) | 30(20,40) | 110(85,175) | 50(30,65) | 70(50,80) |
| 1.225 | 140(85,215) | 80(60,110) | 40(30,45) | 150(110,300) | 50(40,70) | 80(50,115) |
| 1.25 | 170(140,230) | 100(65,130) | 40(30,50) | 165(120,295) | 60(50,85) | 110(55,145) |
| 1.275 | 180(165,300) | 130(80,140) | 50(35,50) | 170(100,290) | 80(60,115) | 140(55,150) |
| 1.3 | 240(170,350) | 140(90,165) | 50(45,80) | - | 110(75,150) | 150(60,200) |
